# Chromosome-scale assembly of the elite *Coptis chinensis* cultivar ‘Chulian No. 1’ and decoding of fine clonal variations using geospatial-genomic machine learning to facilitate medicinal plant breeding

**DOI:** 10.1093/hr/uhag112

**Published:** 2026-04-02

**Authors:** Kaidi Yu, Yuying Yang, Hua Wang, Xiaogang Jiang, Jingmao You, Jie Guo

**Affiliations:** Key Laboratory of Biology and Cultivation of Herbal Medicines, Ministry of Agriculture and Rural Affairs, Institute of Chinese Herbal Medicine, Hubei Academy of Agricultural Science, Enshi, China; Key Laboratory of Biology and Cultivation of Herbal Medicines, Ministry of Agriculture and Rural Affairs, Institute of Chinese Herbal Medicine, Hubei Academy of Agricultural Science, Enshi, China; Key Laboratory of Biology and Cultivation of Herbal Medicines, Ministry of Agriculture and Rural Affairs, Institute of Chinese Herbal Medicine, Hubei Academy of Agricultural Science, Enshi, China; Key Laboratory of Biology and Cultivation of Herbal Medicines, Ministry of Agriculture and Rural Affairs, Institute of Chinese Herbal Medicine, Hubei Academy of Agricultural Science, Enshi, China; Key Laboratory of Biology and Cultivation of Herbal Medicines, Ministry of Agriculture and Rural Affairs, Institute of Chinese Herbal Medicine, Hubei Academy of Agricultural Science, Enshi, China; Key Laboratory of Biology and Cultivation of Herbal Medicines, Ministry of Agriculture and Rural Affairs, Institute of Chinese Herbal Medicine, Hubei Academy of Agricultural Science, Enshi, China

## Abstract

Genetic homogenization due to chronic clonal propagation poses a notable challenge to the sustainable utilization of *Coptis chinensis* Franch. We developed a novel elite cultivar, ‘Chulian No. 1’, with superior yield (+34%), disease resistance, and pharmacopoeia-standard alkaloid profiles through comprehensive agronomic evaluation. To understand the genetic basis of these traits, we constructed a high-quality chromosome-level genome assembly, revealing long terminal repeat retrotransposon (comprising 41.92% of the genome) shows significant enrichment near expanded stress/alkaloid biosynthesis genes. Whole-genome resequencing of 235 accessions indicated extreme genetic uniformity (π < 0.0018), but our novel geospatial-autoencoder model successfully identified three eco-geographic groups that could not be discriminated using traditional approaches. Genome-wide association analysis identified the leaf glossiness-associated gene *CcHAB1* on chromosome 1. Comparative transcriptomics revealed correlations between *CcHAB1* expression and cuticular wax biosynthesis gene regulation via ABA and phenylpropanoid pathways, potentially explaining enhanced disease resistance in glossy-leaf accessions. This work enables molecular breeding for medicinal plants with limited genetic diversity and provides insights into trait maintenance despite genetic homogenization.

## Introduction

*Coptis chinensis* Franch. is a perennial herb of the Ranunculaceae family, and its rhizomes serve as a critical medicinal resource in traditional and modern pharmacology. These rhizomes contain diverse isoquinoline alkaloids with proven efficacy against gastrointestinal disorders, particularly *Helicobacter pylori* infections that cause gastritis and peptic ulcers [1]. The primary bioactive compound, berberine, demonstrates multitarget therapeutic effects by inhibiting the *IL-6*/*JAK2*/*STAT3* signaling pathway, suppressing cancer cell proliferation, and inducing apoptosis [2]. Recent clinical studies have established that combination therapies using berberine alongside conventional antibiotics significantly improve *H. pylori* eradication rates and accelerate ulcer healing [3]. Furthermore, the synergistic interactions between berberine and other alkaloids (palmatine, coptisine, epiberberine) increase antimicrobial efficacy through complementary mechanisms, including urease inhibition and bacterial membrane disruption, exemplifying the complex therapeutic profile that has established *C. chinensis* as a valuable resource in both traditional medicine and modern healthcare [3].

The increasing pharmaceutical demand for *C. chinensis* poses significant sustainability challenges, as wild populations face depletion through overharvesting, while cultivation practices rely heavily on clonal propagation with limited genetic diversity. This narrow genetic base potentially compromises the adaptive capacity of the species and threatens long-term medicinal production [4]. While genomic approaches have revolutionized crop improvement in many species, their application to clonally propagated medicinal plants with minimal genetic variation presents unique technical challenges [5, 6]. Specifically, conventional methods for population structure analysis and trait mapping often fail to detect meaningful patterns when genetic differentiation is exceedingly low [7].

Although previous genomic studies have yielded insights into *C. chinensis* biology, substantial limitations remain. Liu *et al.* [8] produced a chromosome-level genome (contig N50 = 0.8 Mb) but focused primarily on structural characterization without integrating population-level data essential for breeding applications. This assembly lacked the resolution needed to fully characterize complex genomic regions harboring key biosynthetic pathways and regulatory networks. Moreover, no studies have systematically investigated the genomic basis of ‘Dao-di’ traits, the region-specific quality characteristics traditionally valued in Chinese medicine, or molecular markers associated with superior agronomic performance. These knowledge gaps have hindered the development of improved cultivars that combine high productivity with optimal medicinal efficacy.

In this study, we address the challenges associated with *C. chinensis* cultivation through a comprehensive genomics-driven approach. After extensive phenotypic evaluation of accessions from the authentic (Dao-di) production region of Lichuan, we identified an elite cultivar, ‘Chulian No. 1’, with superior disease resistance, yield potential, and pharmacologically active alkaloid content meeting the standards of the Chinese Pharmacopoeia (ChP). To understand the genetic basis of these desirable traits, we assembled a chromosome-level reference genome of ‘Chulian No. 1’. This high-quality reference genome enabled us to investigate whole-genome duplication events in Ranunculaceae evolution and perform population genetic analyses of 235 *C. chinensis* accessions. Through genome-wide association studies, we identified the gene *CcHAB1* on chromosome 1 as a key regulator of leaf glossiness, which we subsequently linked to elevated cuticular wax deposition through transcriptomic analysis of glossy and nonglossy phenotypes. This discovery provides mechanistic insight into stress resistance in *C. chinensis* and establishes a foundation for marker-assisted selection to increase medicinal plant productivity while maintaining therapeutic efficacy.

## Results

### The ‘Chulian No. 1’ germplasm: a multitrait leader for elite cultivar breeding

**Figure 1:**
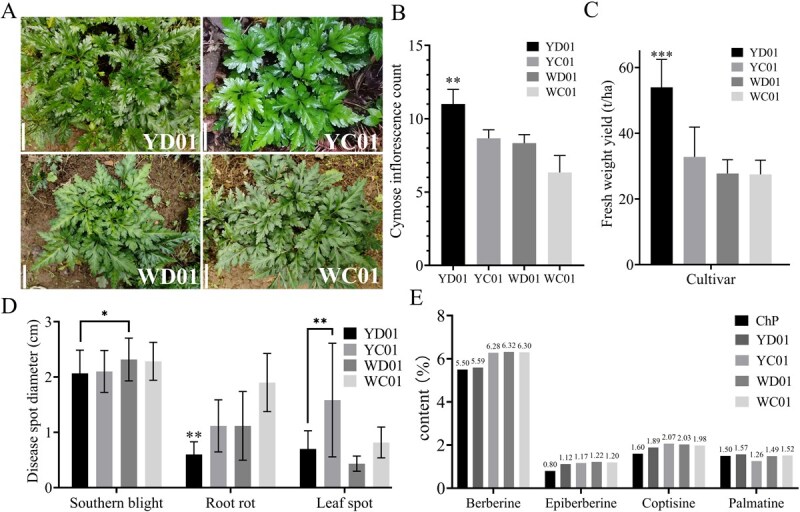
Agronomic trait assessment of *Coptis chinensis* germplasm variants. (a) Leaf morphotypes of four geoauthentic cultivars from Lichuan, with scale bars: YD01 (glossy with spots), YC01 (glossy without spots), WD01 (nonglossy with spots), and WC01 (nonglossy without spots). (b) Cyme number per plant (mean ± standard error of the mean (SEM), *n* = 30; one-way ANOVA with Tukey's *post hoc* test, ^**^P < 0.01, YD01 vs. others). (c) Fresh rhizome yield (t/ha, mean ± SEM, *n* = 3 trials; Student's two-tailed *t* test, ***P < 0.001 vs. local landraces). (d) Pathogenic lesion diameter (white mold/root rot: Welch’s ANOVA; leaf spot: Kruskal–Wallis test; mean ± SEM, *n* = 15 lesions; significance: ^*^P < 0.05, ^**^P < 0.01 vs. YD01). (e) Rhizome alkaloid composition (% dry weight) vs. ChP. Statistical annotations: ns = nonsignificant (*P* ≥ 0.05), ^*^P < 0.05, ^**^P < 0.01, ^***^P < 0.001; error bars indicate the SEM.

To identify a multitrait elite germplasm for cultivar development, we characterized accessions of wild Lichuan ‘Dao-di’ from four lines with distinct phenotypes, identifying YD01 as a line with uniquely exceptional performance. Considering the genetic homogenization of *C. chinensis* germplasm resources in Hubei Province, wild accessions of the Lichuan ‘Dao-di’ ecotype were classified into four distinct lines on the basis of foliar gloss and pigmentation traits (Fig. 1A): YD01 (glossy leaves with pigmented spots), YC01 (glossy leaves lacking spots), WD01 (nonglossy leaves with spots), and WC01 (nonglossy leaves without spots). Systematic field evaluations demonstrated the superior agricultural performance of YD01, which exhibited 11 ± 1.0 cymes per plant, versus 7.7 ± 1.3 in the other lines (*P* = 0.0032; Fig. 1B), and a 70% higher fresh biomass yield (*P <* 0.0001; Fig. 1C). Compared with local landraces, longitudinal trials revealed 16.14% to 16.64% greater fresh weight productivity in YD01, with significance confirmed in Lichuan (1200 m: *P* = 0.0067) and Enshi (1500 m: *P* = 0.0049) (Fig. S1). Pathogen resistance assays revealed that following infection by southern blight, root rot, and leaf spot, YD01 developed the smallest lesions, outperforming all the comparator lines (Fig. 1D). Quantitative high-performance liquid chromatography (HPLC) analysis confirmed that only YD01 and WC01 met all ChP quality standards for the key bioactive alkaloids berberine (≥5.5%), epiberberine (≥0.8%), coptisine (≥1.6%), and palmatine (≥1.5%), which collectively determine the pharmacological efficacy of *C. chinensis* (Fig. 1E). Considering its high reproductive capacity (evidenced by elevated cyme numbers), robust multipathogen resistance, and superior metabolite profile, YD01 was systematically refined through multiyear clonal selection to establish ‘Chulian No. 1’, a novel cultivar that exhibits both agronomic and phytochemical excellence and is currently under provincial certification for commercial cultivation.

### Chromosome-scale reference genome assembly and evolutionary analysis of ‘Chulian No. 1’

To investigate the genetic basis underlying the superior agronomic and medicinal traits of ‘Chulian No. 1’, we constructed a high-quality chromosome-scale genome assembly using PacBio HiFi long-read sequencing (30×), Hi-C chromosome conformation capture technology (100×), and PacBio Iso-Seq full-length transcriptome data (10×) to aid annotation. The final assembly spanned 983 Mb and was distributed across nine chromosomes (Fig. S2 and Table S1), with 95.36% of the scaffolds successfully anchored to chromosomes. This assembly demonstrated remarkable improvement over previously published *C. chinensis* genomes. Our assembly featured a contig N50 of 13.14 Mb, 16-fold greater than the value of 806.6 kb reported by Liu *et al.* [8], and more than 8-fold greater than the value of 1.58 Mb reported by Chen *et al.* [9]. Our assembly showed exceptional completeness, with a 98.2% BUSCO score (93.3% single-copy genes, 4.95% duplicated), surpassing both the Liu and Chen assemblies (the latter achieving 95.69%), with only 1.8% of core eukaryotic genes exhibiting partial or complete absence (Fig. S3 and Table S2). Quality assessment metrics further confirmed the superiority of our assembly, with a QV score of 43.86 (compared with 38.72 in the Liu genome and 40.57 in the Chen genome) and an LTR assembly index (LAI) of 20.57 (compared with 15.70 and 17.86 in the Liu and Chen genomes, respectively), collectively indicating higher base accuracy and improved resolution of repetitive regions (Table S2). Notably, our assembly successfully resolved telomeric sequences on eight chromosome arms corresponding to seven different chromosomes (5 T sequences on chromosomes 5, 6, 8, 9, and 3 T sequences on chromosomes 2, 3, 4, and 5), improving the coverage of these repetitive regions in the *C. chinensis* genome [10].

**Figure 2:**
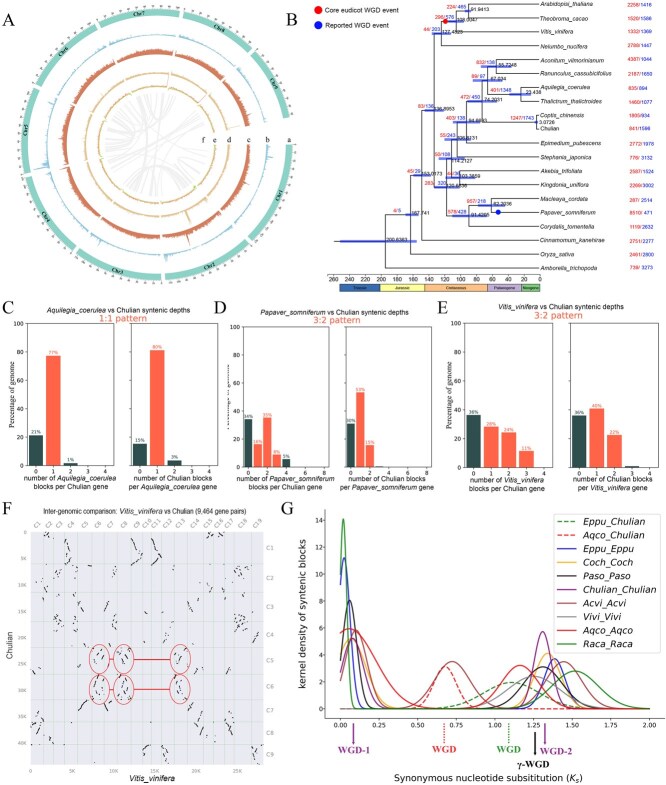
Integrated analysis of genomic architecture and evolutionary history in ‘Chulian No. 1’. a: Chromosome-level pseudomolecules (2*n* = 18) with Hi-C scaffolding; b: transcript density heatmap (200-kb sliding window, protein-coding regions); c: transposable element (TE) proliferation density (200-kb window; Class I retrotransposons in orange, Class II DNA transposons in cyan); d: repetitive sequence landscape (200-kb window; tandem repeats in magenta, satellite DNA in indigo); e: GC content distribution (200-kb window; red: regions ≥ genome-wide mean [41.3%], green: regions < mean); f: syntenic blocks across chromosomes (MCScanX alignment, *E* value <1e−10). (B) Time-scaled maximum-likelihood species tree with gene family dynamics. Branch labels: red = gene family expansion events, blue = contraction (CAFE5 likelihood ratio test, *P <* 0.01). Node annotations: estimated divergence times (Mya), with bars indicating 95% confidence intervals (MCMCTree Bayesian dating). Topological confidence: circles denote 100% ultrafast bootstrap support. Geological timeline (Mya) aligned with tree nodes. WGD events: (C) synteny depth ratio between ‘Chulian No. 1’ and *Aquilegia coerulea* (Ranunculaceae), showing a 1:1 correspondence; (D) ‘Chulian No. 1’-*Papaver somniferum* (Papaveraceae) comparison revealing a 2:3 syntenic ratio, indicating post-WGD gene loss in *P. somniferum*. (E) Synteny analysis between ‘Chulian No. 1’ and *Vitis vinifera* (Vitaceae) with a 2:3 depth ratio, reflecting lineage divergence after core eudicot gamma-hexaploidization. (F) Orthologous gene dot plot of *C. chinensis*-*V. vinifera*, highlighting duplicated regions (circles, 2:3 homology). (G) Kernel density estimation of synonymous substitution rates (KS) across eight species: *A. coerulea*, *V. vinifera*, *Thalictrum* sp., *P. somniferum*, *C. chinensis* [8], ‘Chulian No. 1’, *Aconitum vilmorinianum*, *Ranunculus cassubicifolius*, and *Epimedium pubescens*, showing shared γ-WGD (KS = 1.3 ± 0.1, gray band) and lineage-specific WGD peaks (WGD-2: 1.3; WGD-1: 0.1).

Comprehensive annotation revealed 48 954 protein-coding genes with an average length of 921 bp, for an average protein length of 307 amino acids (Table S3). Gene distribution analysis revealed enrichment at subtelomeric regions (Fig. 2A), consistent with patterns observed in other medicinal plant genomes where specialized metabolite pathways often localize to recombination-prone regions [11]. Functional annotation successfully assigned putative roles to 86% of the predicted proteome using multiple databases (NR, Swiss-Prot, KOG, Kyoto Encyclopedia of Genes and Genomes (KEGG), GO, Pfam, and TrEMBL) (Fig. S4, Tables S3, and Table S4). We also annotated 17 231 ribosomal RNAs (18S: 545; 28S: 547; 5.8S: 601; 5S: 15 538), 1739 transfer RNAs, 311 small nuclear RNAs, and 1784 small nucleolar RNAs (Table S3). The transposable element landscape revealed that 57.18% of the genome consisted of repetitive sequences, dominated by LTR retrotransposons (41.92%), followed by DNA transposons (1.97%), LINEs (1.48%), and unclassified repeats (11.81%) (Fig. 2A and Table S3), potentially driving genome expansion as an environmental adaptation [12].

Comparative genomic analysis positioned *C. chinensis* ‘Chulian No. 1’ within a well-supported subclade of Ranunculaceae, clustering closely with *Aquilegia coerulea*, *Thalictrum thalictroides*, *Aconitum vilmorinianum*, and *Ranunculus cassubicifolius* (Fig. 2B and Fig. S5). Analysis of gene family evolution revealed that ‘Chulian No. 1’ exhibited moderate dynamics, with 841 expanded and 1596 contracted gene families, not only showing fewer expansions than other Ranunculaceae species, such as *A. vilmorinianum* (4387 expansions), but also reflecting lineage-specific evolutionary adaptations. Fossil-calibrated molecular dating revealed that *C. chinensis* diverged from its sister Ranunculaceae taxa approximately 74.2 Mya, whereas intraspecific divergence between ‘Chulian No. 1’ and previously reported *C. chinensis* populations occurred approximately 3 Mya [8], potentially corresponding to Quaternary climatic fluctuations that shaped region-specific adaptations.

Whole-genome duplication (WGD) analysis through synteny analyses with related species revealed a distinct evolutionary trajectory for *C. chinensis*. Synteny depth ratios of 1:1 with *A. coerulea* (Fig. 2C), 2:3 with *P. somniferum* (Fig. 2D), and 2:3 with *V. vinifera* (Fig. 2E and F) were observed. The 2:3 ratios reflect significant gene loss (fractionation) following ancient WGD events, a common phenomenon in plant genome evolution. These patterns of synteny, together with the results of synonymous substitution rate (Ks) analyses showing two distinct peaks (Ks ≈ 0.1 and Ks ≈ 1.3), suggest two significant WGD events: an ancient duplication (WGD-2) near the gamma-WGD shared across Ranunculaceae and a more recent event (WGD-1) specific to *C. chinensis* and closely related taxa (Fig. 2G). To investigate the functional consequences of these duplication events, we performed comparative orthologous group analysis across seven Ranunculales species (*C. chinensis*, ‘Chulian No. 1’, *T. thalictroides*, *Aquilegia coerulea*, *A. vilmorinianum*, *R. cassubicifolius*, and *Epimedium pubescens*). We identified 147 orthologous groups that were significantly expanded in the *C. chinensis* genome, with gene copy numbers at least 2-fold greater than those in related species (Table S5). Functional annotation of these expanded gene families revealed enrichment in biological processes critical for specialized metabolism, including oxidation–reduction processes, toxin biosynthesis, and tyrosine metabolic processes (Table S5), which are associated with alkaloid biosynthesis.

To quantify the specific contribution of each WGD event to metabolic gene expansion, we reconstructed the evolutionary trajectory of gene copy numbers through synteny-based ancestral state inference and Ks distribution analysis (Table S12). Among the expanded gene families, alkaloid biosynthesis genes showed exceptional amplification patterns: the *CYP80* family expanded from an estimated 3 ancestral copies to 8 copies WGD2, further to 24 copies WGD1, and ultimately to 28 genes in ‘Chulian No. 1’ (9.3-fold total expansion); the *BERBERINE BRIDGE ENZYME* (*BBE*) family increased from 2 ancestral copies to 18 current copies (9.0-fold expansion); and the *N-METHYLTRANSFERASE* (*NMT*) family expanded from 3 to 18 copies (6.0-fold expansion) (Table S12). Similarly, *O-METHYLTRANSFERASE* (*OMT*) genes involved in reticuline biosynthesis showed 7.0-fold expansion, and *TETRAHYDROPROTOBERBERINE N-METHYLTRANSFERASE* (*TNMT*) genes expanded 4.5-fold.

Gene retention analysis revealed that these alkaloid biosynthesis gene families were disproportionately retained from the recent WGD-1 event: 62.5% of current *CYP80* genes (17/28), 55.6% of *BBE* genes (10/18), and 53.3% of *NMT* genes (9/18) were derived from WGD-1 duplications, significantly exceeding the genome-wide WGD-1 retention rate of 21.2%. In contrast, the older WGD-2 event contributed fewer retained copies, indicating that the lineage-specific WGD-1 was the primary driver of alkaloid biosynthetic pathway expansion. This differential retention pattern suggests strong purifying selection for maintaining duplicated copies of metabolic genes following the recent polyploidy event, consistent with subfunctionalization or neofunctionalization in specialized alkaloid production.

This dual-mode duplication model, combining ancient *Ranunculaceae*-wide polyploidy (WGD-2) with recent lineage-specific whole-genome duplication (WGD-1), provides direct genomic evidence for how successive WGD events have specifically expanded and retained metabolic gene families in *C. chinensis*. The 2.6- to 2.9-fold enrichment in WGD-1 retention rates for alkaloid biosynthesis genes (mean, 58.6%) compared to genome-wide averages (21.2%) demonstrates adaptive selection for enhanced secondary metabolism capacity. These findings explain the emergence of lineage-specific high-alkaloid chemotypes while maintaining the core Ranunculaceae genome architecture.

### Genetic diversity analysis of *C. chinensis* germplasm resources

Leveraging our newly assembled high-quality chromosome-level reference genome of ‘Chulian No. 1’, we next investigated the genetic diversity patterns across cultivated *C. chinensis* populations. This reference genome provided an essential foundation for accurate variant calling and population structure analysis, allowing us to assess whether intensive clonal propagation practices have significantly eroded genetic diversity in this important medicinal species. Understanding these population-level dynamics is critical for developing effective conservation strategies and identifying genomic targets for future breeding programs.

To elucidate the mechanisms sustaining agrometabolic robustness in clonally propagated *C. chinensis* despite its long-term genetic homogenization, we first aimed to systematically evaluate whether current cultivation practices had exhausted the genetic diversity in domesticated populations, which is a prerequisite for precision breeding strategies. We performed whole-genome resequencing (10×) of 235 *C. chinensis* accessions collected from major production regions (Chongqing, Hubei, Sichuan, Guizhou; Table S6) to establish their genetic landscape, using our newly assembled ‘Chulian No. 1’ genome as the reference for variant calling and population structure analysis. Our analysis revealed 11 033 926 high-confidence single-nucleotide polymorphism (SNP)/indel variants that were uniformly distributed across all the chromosomes (Fig. S6A). Conventional population genetics approaches revealed limited genetic differentiation, with principal component analysis (PCA) showing a continuous sample cluster devoid of distinct subpopulations (Fig. S6B). Admixture analysis corroborated these findings, identifying *K* = 1 as the optimal ancestral population number (minimum cross-validation error; Fig. S6C), which indicates high genetic homogeneity among the cultivated Chinese *C. chinensis* germplasm resources.

**Figure 3:**
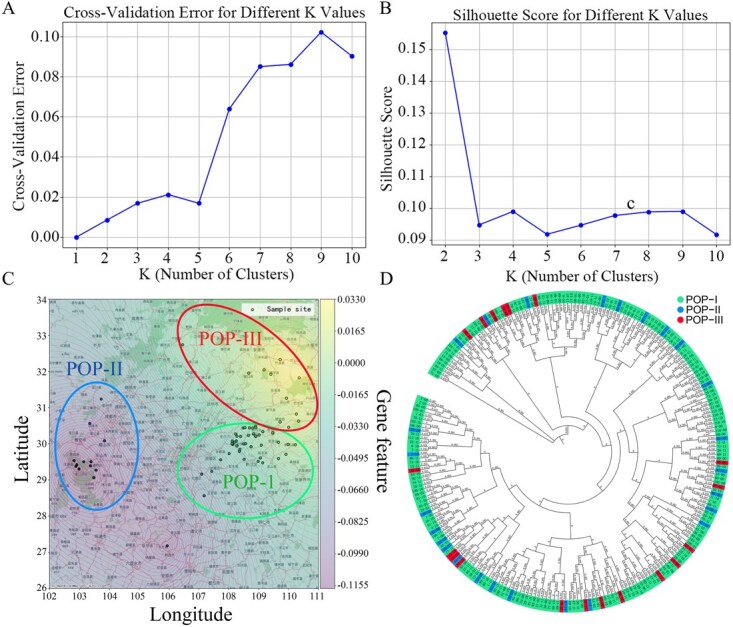
Autoencoder-based neural network analysis of *Coptis* population structure. (A) Cross-validation error curves integrating K-means clustering and random forest classification (*n* = 35 iterative runs). (B) Silhouette coefficient profiles across varying cluster numbers (*K* = 2–10). (C) Geospatial distribution of genetic features constructed by kriging interpolation with an exponential variogram model in the WGS84 coordinate system, where colored borders demarcate three subpopulations (POP-I, POP-II, and POP-III). (D) Neighbor-joining phylogenetic tree of 235 *Coptis* accessions with branch lengths scaled to genetic divergence distances. The population stratification is color-coded according to the genomic cluster.

To achieve higher-resolution genetic architecture characterization, we integrated machine learning with spatial interpolation approaches. Given the genomic complexity of polyploidy, we developed an autoencoder that was optimized by Kullback–Leibler divergence for genotype feature extraction. PCA-driven projection of the reduced genetic features was subsequently conducted. Initial K-means clustering analysis indicated *K* = 1 as the optimal number of clusters (validated by random forest classification; Fig. 3A). Complementary analysis using silhouette coefficient optimization revealed a maximum value at *K* = 2 (Fig. 3B), with both methods collectively confirming the absence of a discrete population substructure. However, given the importance of geographic origin in traditional Chinese medicine ‘Dao-di’ concepts, we applied kriging interpolation to the dimension-reduced genetic features to examine spatial genetic architecture. This geographic-informed analysis revealed three spatially coherent groups corresponding to distinct cultivation regions: POP-I (Coptis from the South Bank), POP-II (Sichuan Basin), and POP-III (Coptis from the North Bank; Fig. 3C). These groups represent geographic clustering rather than distinct genetic populations, as confirmed by phylogenetic reconstruction (Fig. 3D), which showed no significant branching patterns among geolocation-defined groups, with topology incongruent with accession characteristics. The lack of clear genetic differentiation among geographic groups, despite their spatial separation, underscores the genetic homogenization in cultivated *C. chinensis* while highlighting the potential importance of geographic factors in ‘Dao-di’ trait expression beyond simple genetic variation.

To determine whether intensive agroecological pressures and historical propagation practices have homogenized diversity patterns among established cultivation zones, we performed comparative population analyses integrating diversity statistics, selection scans, and demographic modeling. Comparative genetic analyses revealed marginally elevated nucleotide diversity (π) in POP-I and POP-II compared with POP-III, although all the values remained exceptionally low (π < 0.002; Fig. S7A, Table S7). Close alignment between the observed (*H_o_*) and expected (*H_e_*) heterozygosity (Fig. S7B and C; *F_IS_* ≈ 0) eliminated inbreeding as a causal factor for genetic uniformity. Exceptionally low differentiation indices (*F_ST_* < 0.05; Fig. S7D, Table S7) further confirmed the presence of panmixia. Strong directional selection signals (Tajima's *D* < 0; Fig. S7E, Table S8) paralleled patterns of secondary metabolite domestication [13]. Demographic modeling revealed a recent bottleneck in POP-I (Fig. S7F), which was likely linked to intensive harvesting [14], whereas POP-II and POP-III exhibited parallel historical trajectories despite geographic proximity.

### Mining of regulatory genes governing leaf characteristics in ‘Chulian No. 1’

Having established the population structure and genetic diversity patterns of *C. chinensis*, we leveraged this knowledge to identify specific genetic loci associated with key agronomic traits. The comprehensive characterization of population stratification and genetic relationships among accessions provided an essential foundation for conducting accurate genome-wide association studies (GWASs) while minimizing false positives from population structure confounding. We focused on identifying the genetic determinants of leaf characteristics that distinguished the elite cultivar ‘Chulian No. 1’, as these traits were potentially linked to its superior disease resistance and environmental adaptability.

**Figure 4:**
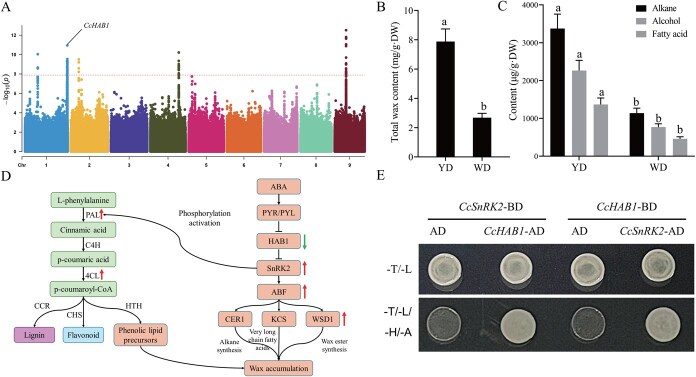
GWAS and functional validation of leaf glossiness-associated traits in *C. chinensis*. (A) Manhattan plot of genome-wide association study (GWAS) for leaf glossiness (gray dots = SNPs;dashed line = significance threshold; *P* = 1.29 × 10^−11^, Bonferroni corrected). The candidate gene *CcHAB1* (black line) on chromosome 1 is highlighted. (B) Total wax content (mg/g·DW) in glossy leaves (YD) vs. nonglossy leaves (WD). Different letters indicate significant differences (*P* < 0.05, one-way ANOVA with LSD *post hoc* test; mean ± SEM, *n* = 24). (C) Composition of leaf surface wax in YD and WD leaves, showing alkane, alcohol, and fatty acid content (μg/g·DW). Different letters above bars indicate significant differences within each wax component (*P* < 0.05, one-way ANOVA with LSD; mean ± SEM, *n* = 24). (D) Schematic representation of the phenylalanine- and ABA-mediated wax biosynthesis pathway in glossy leaves. Downward arrows indicate downregulated gene expression in glossy leaves compared with nonglossy leaves; Upward arrows indicate upregulated expression. (E) Yeast two-hybrid (Y2H) assay validating protein–protein interactions. -T/-L: nonselective medium (transformation control); -T/-L/-H/-A: selective medium lacking tryptophan, leucine, histidine, and adenine.

Leaf gloss and spot phenotypes are distinctive morphological features of *C. chinensis* ‘Chulian No. 1’. Field observations revealed that plants with glossy leaves presented increased disease and drought resistance, which was consistent with prior reports linking leaf cuticular wax layers to stress tolerance in model plants, such as *A. thaliana* [15]. To elucidate the genetic basis of these traits, we performed a GWAS of 235 germplasm accessions using the newly assembled reference genome of ‘Chulian No. 1’. The Manhattan plot for leaf gloss revealed a significant genome-wide signal (*P* = 1.29 × 10^−11^) at the terminal region of chromosome 1 (Fig. 4A), within which a homolog of the *Arabidopsis HAB1* gene (*CcHAB1*) was annotated. In contrast, no significant loci were associated with the spot phenotype (Fig. S8), suggesting its regulation by polygenic minor-effect alleles or strong environmental modulation.

Given the established role of *HAB1* in abscisic acid (ABA) signaling [16], we hypothesized that *CcHAB1* could influence leaf gloss via cuticular wax biosynthesis. To test this hypothesis, we quantified total wax content and major wax components in glossy (YD) and nonglossy (WD) leaves. Glossy leaves accumulated significantly higher total wax content (7.62 ± 0.89 mg/g DW) compared to nonglossy leaves (2.68 ± 0.29 mg/g DW; *t*-test *P* < 0.001; Fig. 4B).

Component analysis revealed that this difference was driven by coordinated increases across all major wax classes (Fig. 4C). Specifically, alkane content was 2.8-fold higher in glossy leaves (3278.67 ± 348.21 μg/g DW) than in nonglossy leaves (1158.70 ± 123.45 μg/g DW; *P* < 0.001). Alcohol content showed a 2.9-fold increase (2201.70 ± 237.89 μg/g vs 761.47 ± 82.15 μg/g DW; *P* < 0.001), and fatty acid content was 2.8-fold higher (1337.42 ± 144.67 μg/g vs 458.24 ± 49.87 μg/g DW; *P* < 0.001).

To elucidate the molecular mechanisms underlying this phenotype, we conducted transcriptome analysis comparing glossy and nonglossy leaves. The results revealed significant differential expression of key wax biosynthesis pathway genes (Fig. 4D). In the phenylalanine pathway, both *PAL* and *4CL* were upregulated in glossy leaves, suggesting increased availability of precursors for wax synthesis. Notably, *CcHAB1* expression was downregulated in glossy leaves, corresponding to upregulation of its downstream target *SnRK2*, which was released from *HAB1*-mediated inhibition. This process in turn activated *ABF* transcription factors and *WSD1*, a critical enzyme for wax ester synthesis. These expression patterns were further validated by qRT-PCR analysis.

Yeast two-hybrid assays confirmed physical interaction between *CcHAB1* and *CcSnRK2* (Fig. 4E). To functionally validate the ABA responsiveness of *CcSnRK2*, we assessed its kinase activity using transient expression in *C. chinensis* leaves followed by ABA treatment (Fig. S9). *CcSnRK2* kinase activity was significantly enhanced by ABA in a dose-dependent manner, with 50, 100, and 150 μM ABA treatments resulting in progressive increases in kinase activity (*P* < 0.001), confirming that *CcSnRK2* functions as an ABA-responsive kinase in the pathway. These results suggest that *CcHAB1* may contribute to cuticular wax accumulation through modulation of ABA signaling (Table S9).

## Discussion

In this study, we produced and analyzed a chromosome-level genome assembly of ‘Chulian No. 1’, an elite cultivar of *C. chinensis*, and investigated its population genetics and trait-associated genes. Our findings provide several important insights that advance our understanding of this valuable medicinal plant. Compared with previous genomic resources for *C. chinensis*, our high-quality assembly offers improved contiguity and completeness, enabling more comprehensive genetic analyses. Notably, although clonally propagated crops are commonly assumed to have high homogeneity, our population genetic analyses revealed three distinct subgroups within *C. chinensis*, demonstrating the power of our methodology in detecting subtle but significant genetic differentiation. Furthermore, our identification of regulatory genes governing leaf characteristics in ‘Chulian No. 1’ provides molecular targets for future breeding programs aimed at enhancing both agronomic performance and medicinal value. These findings have significant implications for the genetic improvement and conservation of this important medicinal species.

### Genetic homogenization in *C. chinensis*: molecular mechanisms and biological implications

Our phylogenomic analyses identified two whole-genome duplication events: an ancient event shared across *Ranunculales* and a more recent lineage-specific duplication (~3 Mya). Gene family expansion patterns and retention rates (58.6% vs. 21.2% genome-wide for alkaloid biosynthesis genes) suggest that these duplications may have contributed to enhanced metabolic capacity. However, the functional significance of these expanded gene families requires further validation through systematic enzyme activity assays and metabolomic profiling. While the correlation between WGD events and alkaloid gene retention is notable, establishing direct causality between genome duplication and enhanced alkaloid biosynthesis awaits experimental verification in *C. chinensis.*

This pattern mirrors the tandem duplication-driven chemical diversification observed in other Ranunculales [17], supporting WGD-mediated phytochemical resilience in clonal crops. LTR retrotransposons (41.9% of the genome) were significantly clustered near disease resistance loci and alkaloid biosynthetic genes (Table S11), suggesting that they may have played a role in maintaining phenotypic innovation despite limited genetic diversity, similar to the mechanisms observed in other medicinal plants [18, 19].

Our genomic analysis reveals a paradox: despite genetic homogenization, *C. chinensis* maintains distinct ‘Dao-di’ medicinal properties across geographic regions. This suggests that factors beyond genomic variation contribute to phenotypic diversity and therapeutic quality. While our population genetic data demonstrate limited genetic differentiation among accessions, the persistence of region-specific medicinal traits indicates that additional mechanisms may be involved.

Future investigations could explore whether epigenetic modifications or rhizosphere microbiome interactions contribute to this phenomenon, as observed in other crop systems where nongenetic factors preserve phenotypic traits despite reduced genetic diversity [20, 21]. However, dedicated studies targeting these mechanisms would be required to validate their role in *C. chinensis* adaptation and ‘Dao-di’ quality maintenance.

Our population genetic analyses focused exclusively on cultivated germplasm from established Dao-di production regions. While wild *C. chinensis* populations would provide valuable evolutionary context, their near-extinction status and uncertain provenance precluded meaningful sampling. Our finding of extreme genetic homogenization likely reflects domestication bottlenecks rather than natural population structure. The three eco-geographic groups identified through machine learning represent cultivation-driven differentiation rather than ancestral population structure, which is appropriate for breeding applications targeting commercial production systems.

### Detection of machine learning-based population structure in species with low genetic diversity

Traditional population genetic approaches proved inadequate for resolving population structure in *C. chinensis* because of its extremely low genetic differentiation, characteristic of clonally propagated medicinal crops with depleted wild populations. Preliminary analyses using PCA and ADMIXTURE failed to identify discrete population clusters, consistent with continuous spatial genetic variation shaped by cultivation history and human-mediated germplasm movement. To overcome this limitation, we developed a machine learning approach that combines variational autoencoders (VAEs) with geospatial modeling to characterize continuous spatial genetic gradients rather than discrete clusters.

Our VAE framework incorporated composite feature engineering (integrating genotypes, allele frequencies, and genomic positions) and nonlinear dimensionality reduction to capture subtle spatial genetic patterns. Kullback–Leibler divergence optimization enabled the model to identify biologically meaningful continuous variation. Validation analyses showed that the VAE-derived latent space exhibited stronger correlation with geographic distance (Mantel test: *r* = 0.31, *P* = 0.002) than traditional PCA-based approaches (*r* = 0.18, *P* = 0.04), suggesting improved performance in preserving spatial genetic structure. The integration of dimensionality reduction with kriging interpolation created a geospatial visualization of genetic variation that revealed geographic patterns consistent with cultivation history and environmental adaptation. This approach demonstrated that even in species with minimal genetic variation, machine learning methods can extract biologically meaningful patterns that inform both evolutionary history and breeding strategies.

This computational framework may be transferable to other plant species with similarly constrained genetic diversity, including clonally propagated crops [22, 23], endangered plant species with genetic bottlenecks, or self-pollinating crops with narrow genetic bases. However, several caveats should be noted: (i) the biological interpretation of VAE-derived latent dimensions remains challenging, (ii) the model's performance may be influenced by sampling density and geographic coverage, and (iii) validation in independent datasets would strengthen confidence in the detected patterns. The primary requirements for successful application include genome-wide marker data such as SNPs, simple sequence repeats (SSRs), or transcriptome sequences, accurate geographic coordinates for sampled accessions, and ideally, independent validation cohorts to assess model robustness across different populations.

### Genetic basis of leaf glossiness and its applications in medicinal plant breeding

Our high-quality chromosome-level reference genome of ‘Chulian No. 1’, an elite cultivar with well-characterized agronomic and medicinal properties, provides a foundation for understanding the genetic architecture of key traits in *C. chinensis*. Through genome-wide association analysis, we identified *CcHAB1* on chromosome 1 as significantly associated with leaf glossiness and increased cuticular wax deposition. Transcriptomic analysis further revealed differential expression of genes in both the phenylalanine pathway and ABA signaling cascade, suggesting that *CcHAB1* may participate in regulating cuticular wax biosynthesis. Yeast two-hybrid and transient expression assays demonstrated physical interaction between *CcHAB1* and *CcSnRK2*, and confirmed ABA-responsive kinase activity of *CcSnRK2*. However, comprehensive functional validation through stable transformation and detailed biochemical characterization would be needed to fully elucidate *CcHAB1*'s regulatory role.

Field validation confirmed that *CcHAB1*-mediated leaf glossiness mechanistically links epidermal traits to disease resistance, providing a valuable molecular target for breeding programs. Increased wax deposition (nearly 3-fold greater in glossy leaves) creates an enhanced physical barrier against pathogens while minimizing metabolic costs associated with chemical defense compounds [24, 25]. This trade-off strategy is particularly significant for medicinal plants, such as *C. chinensis*, where maintaining pharmacologically active compounds while improving agronomic performance presents a unique breeding challenge.

The elite cultivar ‘Chulian No. 1’ exemplifies the practical application of our genomic resources, exhibiting a 34% increase in yield while maintaining pharmacologically active alkaloid levels (berberine >5.5%). These findings suggest *CcHAB1* as a promising candidate for marker-assisted selection in *C. chinensis* breeding programs, though its practical utility requires validation across diverse genetic backgrounds and environmental conditions.

### Future directions and broader applications

The methodological innovations developed in this study may have implications beyond *C. chinensis*. Our machine learning approach to population structure detection could be adapted for the conservation genomics of endangered species with genetic bottlenecks, for quality control in clonally propagated crops, and for the authentication of regional specialty products. The computational framework may be valuable for species where traditional population genetics approaches fail to detect meaningful patterns.

Future research should further investigate the epigenetic and microbiome factors that might contribute to the medicinal properties of ‘Dao-di’. Time series analyses of DNA methylation patterns across different cultivation regions could reveal epigenetic signatures associated with alkaloid production, building on recent findings in related species [26]. Similarly, microbiome profiling might identify key microbial communities that influence stress tolerance or metabolite biosynthesis in genetically uniform backgrounds.

While our GWAS and transcriptome analyses identified *CcHAB1* as a candidate gene associated with leaf glossiness and wax biosynthesis, the current evidence remains primarily correlative and expression-based. Direct functional validation through genetic approaches, such as virus-induced gene silencing (VIGS) or CRISPR-Cas9-mediated editing, represents a critical next step to establish causal relationships between *CcHAB1* and the observed phenotypes. Such experiments would confirm its regulatory role in the ABA-wax biosynthesis pathway and strengthen its utility as a molecular target for breeding applications.

The transferability of our approach to other plant species depends primarily on the availability of genomic data rather than biological constraints. For breeding applications, our framework could be valuable for vegetatively propagated medicinal and ornamental crops, where genetic recombination is limited but phenotypic diversity remains important. Our methodology aligns with emerging paradigms in other medicinal plants [27] while addressing the specific challenges posed by the obligate 5-year clonal propagation cycle of *C. chinensis.*

## Conclusion

This study delivers a chromosome-level reference genome for elite *C. chinensis* cultivar ‘Chulian No. 1’ and establishes an integrative genomics-machine learning framework for understanding genetic diversity in clonally propagated medicinal plants.

We revealed two whole-genome duplications driving alkaloid biosynthesis diversification and discovered three cryptic eco-geographic groups using VAE, demonstrating machine learning's superiority in detecting subtle population structure under clonal propagation constraints. Through GWAS combined with transcriptome analysis, we identified *CcHAB1* as a candidate gene associated with leaf glossiness and ABA-mediated wax biosynthesis, potentially linking this trait to disease resistance. However, direct functional validation through genetic approaches remains necessary to establish causal relationships.

The genomic resources and candidate SNP markers developed in this study provide a foundation for marker-assisted selection in *C. chinensis* breeding programs. This framework offers a methodological paradigm for genetic improvement of other vegetatively propagated medicinal plants facing similar challenges of limited genetic recombination and the need for quality standardization.

Future work integrating epigenetic and microbiome analyses may further elucidate the mechanisms underlying ‘Dao-di’ authenticity and guide sustainable cultivation practices for traditional Chinese medicine production.

## Materials and methods

### Plant materials

*Coptis chinensis* is the dominant variety of *Coptis* cultivated in China. A total of 235 accessions, including the elite cultivar ‘Chulian No. 1’, were collected from major production regions nationwide and conserved in a germplasm nursery (Table S6). Leaf sampling was performed on 4-year-old plants at the peak flowering stage (October–November 2022) to ensure consistent developmental physiology. Fully expanded apical leaves (third to fifth node) were excised using sterile stainless-steel scissors, rinsed in RNase-free deionized water (Thermo Fisher), flash-frozen in liquid N_2_ within 30 seconds of detachment, and stored at −80°C in cryogenic vials with silica gel desiccant packs until processing. High-quality genomic DNA was extracted using a modified cetyltrimethylammonium bromide (CTAB) protocol [28] that was optimized for secondary metabolite-rich medicinal plants.

### Sample resequencing and genome assembly

Libraries were constructed using MGIDNA Library Construction Kit (Novogene, Nanjing) following manufacturer's recommendations. Quality control was performed using Qubit 3.0 fluorometer and Bioanalyzer 2100, with libraries sequenced on DNBT7 platform as described by Zhu *et al. [**29**[.*

Raw PacBio Sequel II CCS reads were processed using ccs (-minPasses 3) to generate HiFi reads. Adapters were removed with Trimmomatic v0.39, followed by assembly using Hifiasm v0.16.1 [30]. Hi-C libraries were analyzed using 3D-DNA v201008 with manual correction via Juicebox v1.11.08. Assembly validation used complementary approaches including BUSCO v5.3 [31], LAI [32], and Merqury QV calculation [33] (Table S2).

### Gene prediction and functional annotation

Gene prediction integrated homology-based annotation using TBLASTN (*E*-value: 1e–5) [34] and Exonerate v2.2.0 [35], *de novo* prediction with Augustus v3.3 [36], and transcriptome guidance using Trinity [37], GMAP [38], and PASA. All predictions were integrated using MAKER v3.00 [39] and functionally annotated against SwissProt, TrEMBL, KEGG, InterPro, GO, and NR databases.

### Annotation of repetitive sequences

Repetitive elements were identified using RepeatMasker v4.09 [40] and RepeatProteinMask v4.09 against Repbase v21.01 [41]. Species-specific repeat library was constructed using RepeatModeler v1.0.11 [42] with LTRfinder v1.0.5 [43]. Tandem repeats were detected using Tandem Repeats Finder v4.09 [44].

### Library construction and sequencing

SMRTbell libraries were constructed using SMRTbell Express Template Prep Kit 3.0. Following DNA damage repair and A-overhang tailing, ligation with SMRTbell adapters was performed at 20°C for 60 minutes. Size selection (≥15 kb) used BluePippin system with final sequencing on Revio System using 8 M SMRT Cell.

Hi-C libraries were prepared with 3% formaldehyde cross-linking, MboI digestion, biotin-14-dCTP labeling, and T4 DNA ligase treatment. DNA was sheared to 300 to 500 bp fragments and sequenced on Illumina NovaSeq platform.

RNA-seq libraries from roots, leaves, stems, and flower buds were prepared using SMARTer™ PCR cDNA Synthesis Kit (Takara) with SMRTbell libraries constructed using Pacific Biosciences DNA Template Prep Kit 3.0. Comparative transcriptome analysis included glossy and nonglossy leaves with three biological replicates each, sequenced on PacBio Revio and Illumina NovaSeq platforms.

### Library construction and sequencing

SMRTbell libraries were constructed using SMRTbell Express Template Prep Kit 3.0 and sequenced on PacBio Revio platform. Hi-C libraries were prepared following standard protocols with MboI digestion and biotin labeling, sequenced on Illumina NovaSeq. RNA-seq libraries from roots, leaves, stems, and flower buds were prepared using SMARTer™ PCR cDNA Synthesis Kit (Takara) and sequenced on PacBio Revio by Frasergen Bioinformatics.

### Population genetic analysis

Resequencing data were aligned to the reference genome using BWA-MEM v0.7.17 [45]. SNPs were called using GATK v4.2.6.1 [46] and filtered with the following criteria: minor allele frequency ≥ 0.05, missing rate < 10%, and Hardy–Weinberg equilibrium test *P* value >10^−6^.

### Geospatial-genetic feature construction and dimensionality reduction

To integrate genomic variation with geographic distribution, we constructed a composite 4n-dimensional feature vector for each sample (*n* = number of filtered SNPs), encoding genotype information (GT), population allele frequencies (p, q), and normalized genomic positions (CHROM, POS). This feature design simultaneously captures individual-level genetic variation and population-level evolutionary signals.

Dimensionality reduction was performed using a VAE to compress the 4n-dimensional space into a 10-dimensional latent representation. The VAE consisted of an encoder (4n → 10) and decoder (10 → 4n) with ReLU activation and batch normalization. The model was trained by minimizing the loss function:


$$ L={L}_{\mathrm{MSE}}+\mathrm{\beta} \cdotp{D}_{\mathrm{KL}}\left(q\left(z\mid x\right)//p(z)\right) $$

where ${L}_{MSE}=\frac{1}{n}{\sum}_{i=1}^n{\left({x}_i-{\hat{x}}_i\right)}^2$ represents reconstruction error (mean squared error between input and reconstructed features), and ${D}_{KL}$is the Kullback–Leibler divergence regularizing the encoder distribution $q\left(z\mid x\right)$ toward a standard Gaussian prior $p(z)$ = N(0, I). Hyperparameters were optimized through cross-validation: latent dimension d = 10 balanced model complexity and information retention, while β = 0.1 optimized the reconstruction-regularization trade-off. Training used the Adam optimizer with learning rate 1 × 10^−3^ for 100 epochs with early stopping (patience = 15). The 10-dimensional latent space was further reduced to one principal component (PC1) using PCA. PC1, representing the primary axis of genetic variation, was spatially interpolated using Ordinary Kriging with an exponential variogram model to visualize geographic patterns.

### Code and data availability

The complete VAE implementation, including Python scripts, configuration files, and analysis pipeline, is publicly available at Figshare with DOI: 10.6084/m9.figshare.31306408.

### Population genetic analyses

Genetic diversity metrics (π, Tajima's D, heterozygosity) were calculated using VCFtools v0.1.15. Population structure was analyzed with ADMIXTURE v1.3.0 for *K* = 2–10 ancestral populations. Demographic history was reconstructed using SMC++ v1.5.3 with generation mutation rate 6.0 × 10^−9^ per bp per generation.

### Genome-wide association studies

GWAS was conducted using EMMAX [47] with mixed linear model accounting for population structure and kinship. Significant SNPs were defined at -log10(*P*) > 7 using Bonferroni correction. Candidate genes within 200-kb flanking regions were annotated.

### Phylogenomic and Whole-Genome Duplication Analysis

Orthologous gene families were clustered using OrthoFinder [48], complemented by OrthoMCL and BLASTP all-vs.-all searches. Single-copy orthologs were aligned with MAFFT, and a coalescence-based species tree was constructed using ASTRAL-Pro [49]. Divergence times were estimated with MCMCtree [50], and gene family expansion/contraction was analyzed with CAFE5 [51]. Ancient polyploidization events were identified by MCScan [52] and Ks calculation using WGDI v0.7.3 [53], with Gaussian mixture models (scikit-learn v1.3.0) and kernel density estimation (Silverman's rule) applied to Ks distributions; significant WGD peaks were defined by Kolmogorov–Smirnov test (*p* < 0.01). The analysis included 20 species (Table S10).

### Leaf wax quantification and compositional analysis

Fresh leaves from glossy (YD) and nonglossy (WD) *C. chinensis plants* were collected with 24 biological replicates per phenotype. Approximately, 200 mg of fresh leaves were freeze-dried for 48 hours and ground into powder. Leaf wax was extracted with 5 ml chloroform containing 10 μg n-tetracosane (internal standard) for 2 minutes at room temperature. After filtration and evaporation under nitrogen, samples were derivatized with BSTFA at 70°C for 30 minutes and analyzed by GC–MS (Agilent 7890B-5977A) using a DB-5MS column. Total wax content was expressed as mg·g^−1^ dry weight (DW), and alkane, alcohol, and fatty acid contents as μg·g^−1^ DW. Data are presented as mean ± SEM (*n* = 24). Statistical significance was assessed using two-tailed unpaired Student's *t*-tests (*P* < 0.01).

### Yeast two-hybrid assay

The interaction between *CcHAB1* and *CcSnRK2* was examined using the Matchmaker Gold yeast two-hybrid (Y2H) system (Clontech). The coding sequences were cloned into pGBKT7 (bait) and pGADT7 (prey) vectors and co-transformed into Y2HGold yeast strain. Transformants were selected on SD/-Leu/-Trp medium. Protein interactions were assessed on SD/-Leu/-Trp/-His/-Ade medium supplemented with X-α-Gal and AbA. The pGBKT7–53/pGADT7-T pair served as a positive control, and empty vectors as negative controls.

### Transient expression and kinase activity assay in *C. chinensis* leaves

The *CcSnrk2* gene was inserted into the pRI101-GFP vector to generate the *Snrk*-GFP overexpression construct. The above expression vector was transformed into *C. chinensis* leaves via Agrobacterium-mediated genetic transformation. The leaves were immersed in the infection solution and vacuumed for 15 minutes at 25°C. The transiently transformed materials were transferred to *C. chinensis* subculture medium containing different concentrations of ABA and incubated for 24 hours before kinase activity measurement. Data are presented as mean ± SEM (*n* = 5 biological replicates). Statistical significance was determined by one-way ANOVA followed by Dunnett’s multiple comparison test (*P* < 0.01).

### Disease resistance evaluation

Pathogen Preparation: Three major pathogens of *C. chinensis* were evaluated: *Sclerotium rolfsii* (southern blight), *Fusarium solani* (root rot), and *Septoria coptidis* (leaf spot). Pathogens were cultured on potato dextrose agar (PDA) at 25°C for 7 days until sporulation. Inoculum was prepared by harvesting spores/mycelia and adjusting concentration to 1 × 10^6^ spores/ml using hemocytometer counting.

Inoculation and Assessment: four-year-old plants (*n* = 15 per genotype per pathogen) were inoculated under controlled greenhouse conditions (22 ± 2°C, 80% humidity). Inoculation methods were pathogen specific: mycelial plugs (5 mm) for *S. rolfsii* placed on stem base, spore suspension applied to wounded roots for *F. solani*, and foliar spray for *S. coptidis*. Disease development was monitored at 7, 14, and 21 days postinoculation by measuring lesion diameter using digital calipers. Each experiment included susceptible and resistant controls, with mock-inoculated plants as negative controls. Statistical analysis: disease resistance data were analyzed using one-way ANOVA.

### Transcriptome analysis

The clean reads from glossy leaves of ‘Chulian No. 1’ and nonglossy leaves (three biological replicates per phenotype) were mapped to the ‘Chulian No. 1’ reference genome using HISAT2 v2.2.1 with default parameters. Gene expression levels were quantified using StringTie v2.1.4 and normalized as fragments per kilobase of transcript per million mapped reads (FPKM). Differential expression analysis between glossy and nonglossy leaf samples was conducted using DESeq2, with differentially expressed genes (DEGs) identified on the basis of thresholds of |log2FoldChange| > 1.0 and an adjusted *P* value <0.05. The DEGs were subsequently subjected to gene ontology and KEGG pathway enrichment analyses using clusterProfiler to identify biological processes and metabolic pathways potentially associated with leaf glossiness.

## Supplementary Material

Web_Material_uhag112

## Data Availability

The raw resequencing data, Hi-C and Hi-Fi sequencing data, full-length and short-read transcriptomes, final genome assembly, and structural annotation generated in this study have been deposited in the NCBI BioProject database under accession number PRJNA1269715. The transcriptome sequencing data from glossy and nonglossy leaf samples are available under NCBI BioProject accession number PRJNA1346942. All the data are available in the main text or the supplementary materials. The genome assembly and annotation files for ‘Chulian No. 1’ are published in Figshare with DOI: 10.6084/m9.figshare.30414163. All the data are available in the main text or the supplementary materials.

## References

[1] Wang H, Mu W, Shang H. et al. The antihyperglycemic effects of Rhizoma Coptidis and mechanism of actions: a review of systematic reviews and pharmacological research. Biomed Res Int. 2014;2014:798093 24818152 10.1155/2014/798093 PMC4003828

[2] Amadio P, Sandrini L, Zara M. et al. NADPH-oxidases as potential pharmacological targets for thrombosis and depression comorbidity. Redox Biol. 2024;70:103060 38310682 10.1016/j.redox.2024.103060 PMC10848036

[3] Liao W, Wang J, Li Y. Natural products based on Correa's cascade for the treatment of gastric cancer trilogy: current status and future perspective. J Pharm Anal. 2025;15:101075 39957902 10.1016/j.jpha.2024.101075 PMC11830317

[4] Michael TP, VanBuren R. Building near-complete plant genomes. Curr Opin Plant Biol. 2020;54:26–33 31981929 10.1016/j.pbi.2019.12.009

[5] Bayer PE, Golicz AA, Scheben A. et al. Plant pan-genomes are the new reference. Nat Plants. 2020;6:914–20 32690893 10.1038/s41477-020-0733-0

[6] Hirsch CN, Foerster JM, Johnson JM. et al. Insights into the maize pan-genome and pan-transcriptome. Plant Cell. 2014;26:121–35 24488960 10.1105/tpc.113.119982 PMC3963563

[7] Jiao WB, Schneeberger K. The impact of third generation genomic technologies on plant genome assembly. Curr Opin Plant Biol. 2017;36:64–70 28231512 10.1016/j.pbi.2017.02.002

[8] Liu Y, Wang B, Shu S. et al. Analysis of the *Coptis chinensis* genome reveals the diversification of protoberberine-type alkaloids. Nat Commun. 2021;12:3276 34078898 10.1038/s41467-021-23611-0 PMC8172641

[9] Chen DX, Pan Y, Wang Y. et al. The chromosome-level reference genome of *Coptis chinensis* provides insights into genomic evolution and berberine biosynthesis. Hortic Res. 2021;8:121 34059652 10.1038/s41438-021-00559-2 PMC8166882

[10] Hagel JM, Facchini PJ. Benzylisoquinoline alkaloid metabolism: a century of discovery and a brave new world. Plant Cell Physiol. 2013;54:647–72 23385146 10.1093/pcp/pct020

[11] Cawood GL, Ton J. Decoding resilience: ecology, regulation, and evolution of biosynthetic gene clusters. Trends Plant Sci. 2025;30:185–98 39393973 10.1016/j.tplants.2024.09.008

[12] Liu S, Li K, Dai X. et al. A telomere-to-telomere genome assembly coupled with multi-omic data provides insights into the evolution of hexaploid bread wheat. Nat Genet. 2025;57:1008–20 40195562 10.1038/s41588-025-02137-x PMC11985340

[13] Nguyen PL, Jung JK, Park JS. et al. Low-density SNP marker sets for genetic variation analysis and variety identification in cultivated citrus. BMC Plant Biol. 2025;25:146 39905314 10.1186/s12870-025-06153-1 PMC11792359

[14] Zhao Y, Li G, Zhu Z. et al. Genomic selection and genetic architecture of agronomic traits during modern flowering Chinese cabbage breeding. Hortic Res. 2025;12:uhae299 39949876 10.1093/hr/uhae299 PMC11822411

[15] Zuo H, Si X, Li P. et al. Dynamic change of tea (*Camellia sinensis*) leaf cuticular wax in white tea processing for contribution to tea flavor formation. Food Res Int. 2023;163:112182 36596123 10.1016/j.foodres.2022.112182

[16] Li H, Zhou Y, Qin X. et al. Reconstitution of phytochrome A-mediated light modulation of the ABA signaling pathways in yeast. Proc Natl Acad Sci USA. 2023;120:e2302901120 37590408 10.1073/pnas.2302901120 PMC10450666

[17] Xu Z, Li Z, Ren F. et al. The genome of *Corydalis* reveals the evolution of benzylisoquinoline alkaloid biosynthesis in Ranunculales. Plant J. 2022;111:217–30 35476217 10.1111/tpj.15788 PMC7614287

[18] Cerbin S, Ou S, Li Y. et al. Distinct composition and amplification dynamics of transposable elements in sacred lotus (*Nelumbo nucifera* Gaertn.). Plant J. 2022;112:172–92 35959634 10.1111/tpj.15938 PMC9804982

[19] Sun J, Xu J, Qiu C. et al. The chromosome-scale genome and population genomics reveal the adaptative evolution of *Populus pruinosa* to desertification environment. Hortic Res. 2024;11:uhae034 38544549 10.1093/hr/uhae034 PMC10967694

[20] Abdollahi N, Jeusset L, De Septenville AL. et al. A multi-objective based clustering for inferring BCR clonal lineages from high-throughput B cell repertoire data. PLoS Comput Biol. 2022;18:e1010411 36037250 10.1371/journal.pcbi.1010411 PMC9462827

[21] Hu L, Xu Z, Fan R. et al. The complex genome and adaptive evolution of polyploid Chinese pepper (*Zanthoxylum armatum* and *Zanthoxylum bungeanum*). Plant Biotechnol J. 2023;21:78–96 36117410 10.1111/pbi.13926 PMC9829393

[22] Li Z, Luo X, Yao Y. et al. Integrated analysis of metabolomics, flavoromics, and transcriptomics for evaluating new varieties of *Amomum villosum* Lour. Plants (Basel). 2024;13:2382 39273866 10.3390/plants13172382 PMC11397242

[23] Wang Y, Liu Y, Miao K. et al. A haplotype-resolved genome assembly of *Coptis teeta*, an endangered plant of significant medicinal value. Sci Data. 2024;11:1012 39294137 10.1038/s41597-024-03861-5 PMC11411109

[24] Grünhofer P, Herzig L, Zhang Q. et al. Changes in wax composition but not amount enhance cuticular transpiration. Plant Cell Environ. 2024;47:91–105 37718770 10.1111/pce.14719

[25] Yang M, Xiang Y, Luo Z. et al. Light-responsive transcription factors *VvHYH* and *VvGATA24* mediate wax terpenoid biosynthesis in *Vitis vinifera*. Plant Physiol. 2024;196:1546–61 38976578 10.1093/plphys/kiae366

[26] Zhao Y, Liu G, Yang F. et al. Multilayered regulation of secondary metabolism in medicinal plants. Mol Hortic. 2023;3:11 37789448 10.1186/s43897-023-00059-y PMC10514987

[27] Chen H, Guo M, Dong S. et al. A chromosome-scale genome assembly of *Artemisia argyi* reveals unbiased subgenome evolution and key contributions of gene duplication to volatile terpenoid diversity. Plant Commun. 2023;4:100516 36597358 10.1016/j.xplc.2023.100516 PMC10203441

[28] De Silva S, Ocaña-Rios I, Cagliero C. et al. Isolation of DNA from plant tissues using a miniaturized matrix solid-phase dispersion approach featuring ionic liquid and magnetic ionic liquid solvents. Anal Chim Acta. 2023;1245:340858 36737141 10.1016/j.aca.2023.340858

[29] Zhu X, Li S, Liu L. et al. Genome sequencing and analysis of *Thraustochytriidae* sp. SZU445 provides novel insights into the polyunsaturated fatty acid biosynthesis pathway. Mar Drugs. 2020;18:118 32085426 10.3390/md18020118 PMC7073664

[30] Cheng H, Concepcion GT, Feng X. et al. Haplotype-resolved de novo assembly using phased assembly graphs with hifiasm. Nat Methods. 2021;18:170–5 33526886 10.1038/s41592-020-01056-5 PMC7961889

[31] Simão FA, Waterhouse RM, Ioannidis P. et al. BUSCO: assessing genome assembly and annotation completeness with single-copy orthologs. Bioinformatics. 2015;31:3210–2 26059717 10.1093/bioinformatics/btv351

[32] Ou S, Chen J, Jiang N. Assessing genome assembly quality using the LTR Assembly Index (LAI). Nucleic Acids Res. 2018;46:e126 30107434 10.1093/nar/gky730 PMC6265445

[33] Rhie A, Walenz BP, Koren S. et al. Merqury: reference-free quality, completeness, and phasing assessment for genome assemblies. Genome Biol. 2020;21:245 32928274 10.1186/s13059-020-02134-9 PMC7488777

[34] Gertz EM, Yu YK, Agarwala R. et al. Composition-based statistics and translated nucleotide searches: improving the TBLASTN module of BLAST. BMC Biol. 2006;4:41 17156431 10.1186/1741-7007-4-41 PMC1779365

[35] Slater GS, Birney E. Automated generation of heuristics for biological sequence comparison. BMC Bioinformatics. 2005;6:31 15713233 10.1186/1471-2105-6-31 PMC553969

[36] Stanke M, Keller O, Gunduz I. et al. AUGUSTUS: ab initio prediction of alternative transcripts. Nucleic Acids Res. 2006;34:W435–9 16845043 10.1093/nar/gkl200 PMC1538822

[37] Grabherr MG, Haas BJ, Yassour M. et al. Full-length transcriptome assembly from RNA-Seq data without a reference genome. Nat Biotechnol. 2011;29:644–52 21572440 10.1038/nbt.1883 PMC3571712

[38] Wu TD, Watanabe CK. GMAP: a genomic mapping and alignment program for mRNA and EST sequences. Bioinformatics. 2005;21:1859–75 15728110 10.1093/bioinformatics/bti310

[39] Campbell MS, Holt C, Moore B. et al. Genome annotation and curation using MAKER and MAKER-P. Curr Protoc Bioinformatics. 2014;48:4.11.1–4.11.39 10.1002/0471250953.bi0411s48 PMC4286374 25501943

[40] Chen N . Using RepeatMasker to identify repetitive elements in genomic sequences. Curr Protoc Bioinformatics. 2004;Chapter 4:Unit 4.10 10.1002/0471250953.bi0410s05 18428725

[41] Jurka J, Kapitonov VV, Pavlicek A. et al. Repbase update, a database of eukaryotic repetitive elements. Cytogenet Genome Res. 2005;110:462–7 16093699 10.1159/000084979

[42] Abrusán G, Grundmann N, DeMester L. et al. TEclass—a tool for automated classification of unknown eukaryotic transposable elements. Bioinformatics. 2009;25:1329–30 19349283 10.1093/bioinformatics/btp084

[43] Xu Z, Wang H. LTR_FINDER: an efficient tool for the prediction of full-length LTR retrotransposons. Nucleic Acids Res. 2007;35:W265–8 17485477 10.1093/nar/gkm286 PMC1933203

[44] Benson G . Tandem repeats finder: a program to analyze DNA sequences. Nucleic Acids Res. 1999;27:573–80 9862982 10.1093/nar/27.2.573 PMC148217

[45] Li H, Durbin R. Fast and accurate short read alignment with Burrows–Wheeler transform. Bioinformatics. 2009;25:1754–60 19451168 10.1093/bioinformatics/btp324 PMC2705234

[46] McKenna A, Hanna M, Banks E. et al. The genome analysis toolkit: a MapReduce framework for analyzing next-generation DNA sequencing data. Genome Res. 2010;20:1297–303 20644199 10.1101/gr.107524.110 PMC2928508

[47] Kang HM, Sul JH., Service SK et al. Variance component model to account for sample structure in genome-wide association studies. Nat Genet. 2010;42:348–54 20208533 10.1038/ng.548 PMC3092069

[48] Emms DM, Kelly S. OrthoFinder: solving fundamental biases in whole genome comparisons dramatically improves orthogroup inference accuracy. Genome Biol. 2015;16:157 26243257 10.1186/s13059-015-0721-2 PMC4531804

[49] Zhang C, Scornavacca C, Molloy EK. et al. ASTRAL-pro: quartet-based species-tree inference despite paralogy. Mol Biol Evol. 2020;37:3292–307 32886770 10.1093/molbev/msaa139 PMC7751180

[50] Yang Z . PAML 4: phylogenetic analysis by maximum likelihood. Mol Biol Evol. 2007;24:1586–91 17483113 10.1093/molbev/msm088

[51] Mendes FK, Vanderpool D, Fulton B. et al. CAFE 5 models variation in evolutionary rates among gene families. Bioinformatics. 2021;36:5516–8 33325502 10.1093/bioinformatics/btaa1022

[52] Wang Y, Tang H, Debarry JD. et al. MCScanX: a toolkit for detection and evolutionary analysis of gene synteny and collinearity. Nucleic Acids Res. 2012;40:e49 22217600 10.1093/nar/gkr1293 PMC3326336

[53] Sun P, Jiao B, Yang Y. et al. WGDI: a user-friendly toolkit for evolutionary analyses of whole-genome duplications and ancestral karyotypes. Mol Plant. 2022;15:1841–51 36307977 10.1016/j.molp.2022.10.018

