# Supplementary material for: Chromosome-scale assembly of the elite Coptis chinensis cultivar ‘Chulian No. 1’ and decoding of fine clonal variations using geospatial-genomic machine learning to facilitate medicinal plant breeding
Source: Hortic Res. 2026 Apr 2;13(8):uhag112. doi: 10.1093/hr/uhag112 (PMC13394188; doi:10.1093/hr/uhag112)
Supplement: Web_Material_uhag112 [file web_material_uhag112.zip › Supplement figure.docx]

**Fig. S1 Yield comparison of YD01 with local cultivars in Enshi and Lichuan**. Fresh rhizome weight per plant (t/ha, **±**SEM).

**Fig. S2 Heat map of Hi-C interaction between the 9 chromosomes of *Coptis chinensis* genome.**


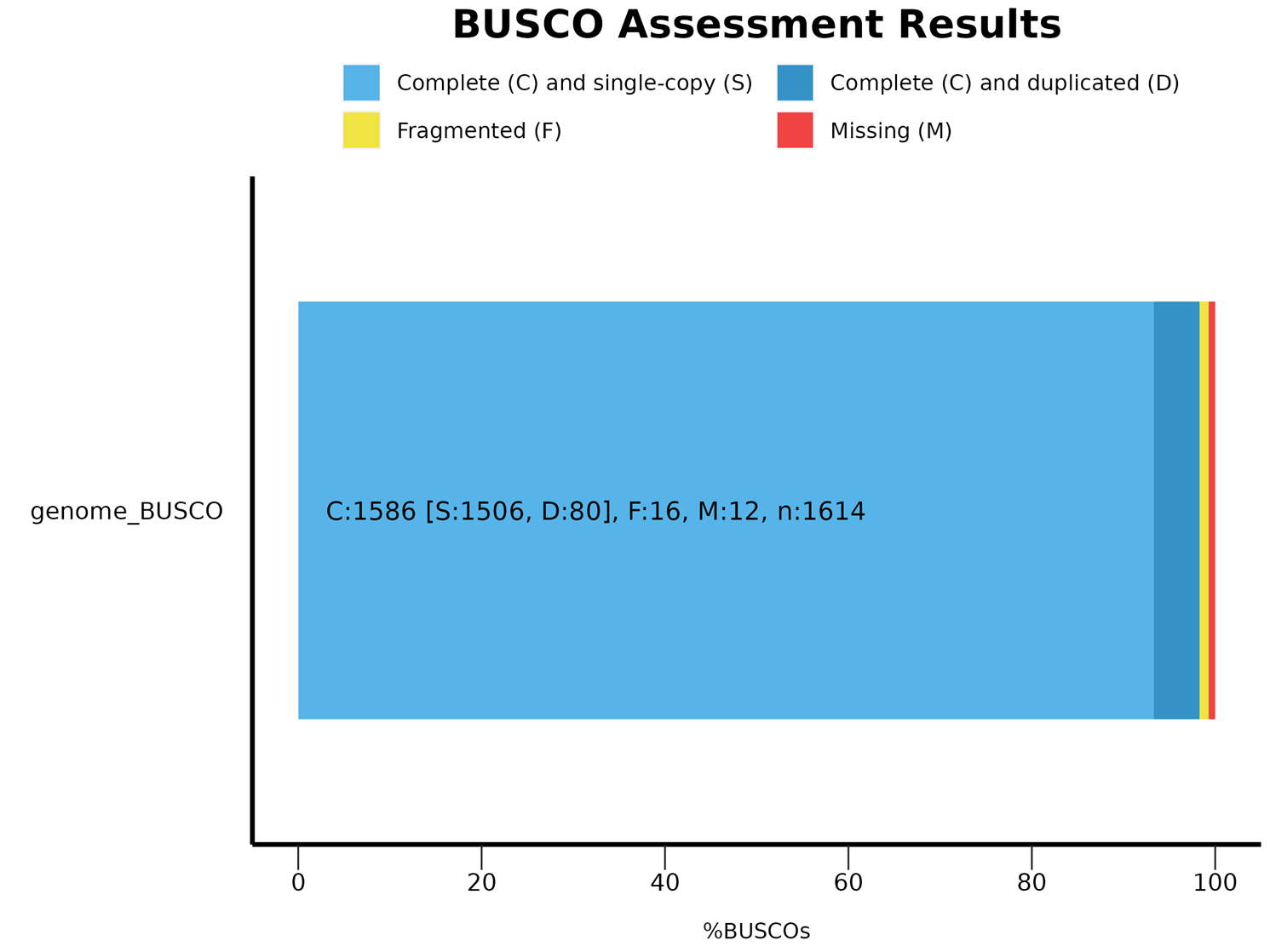


**Fig. S3 Genomic BUSCO evaluation map**


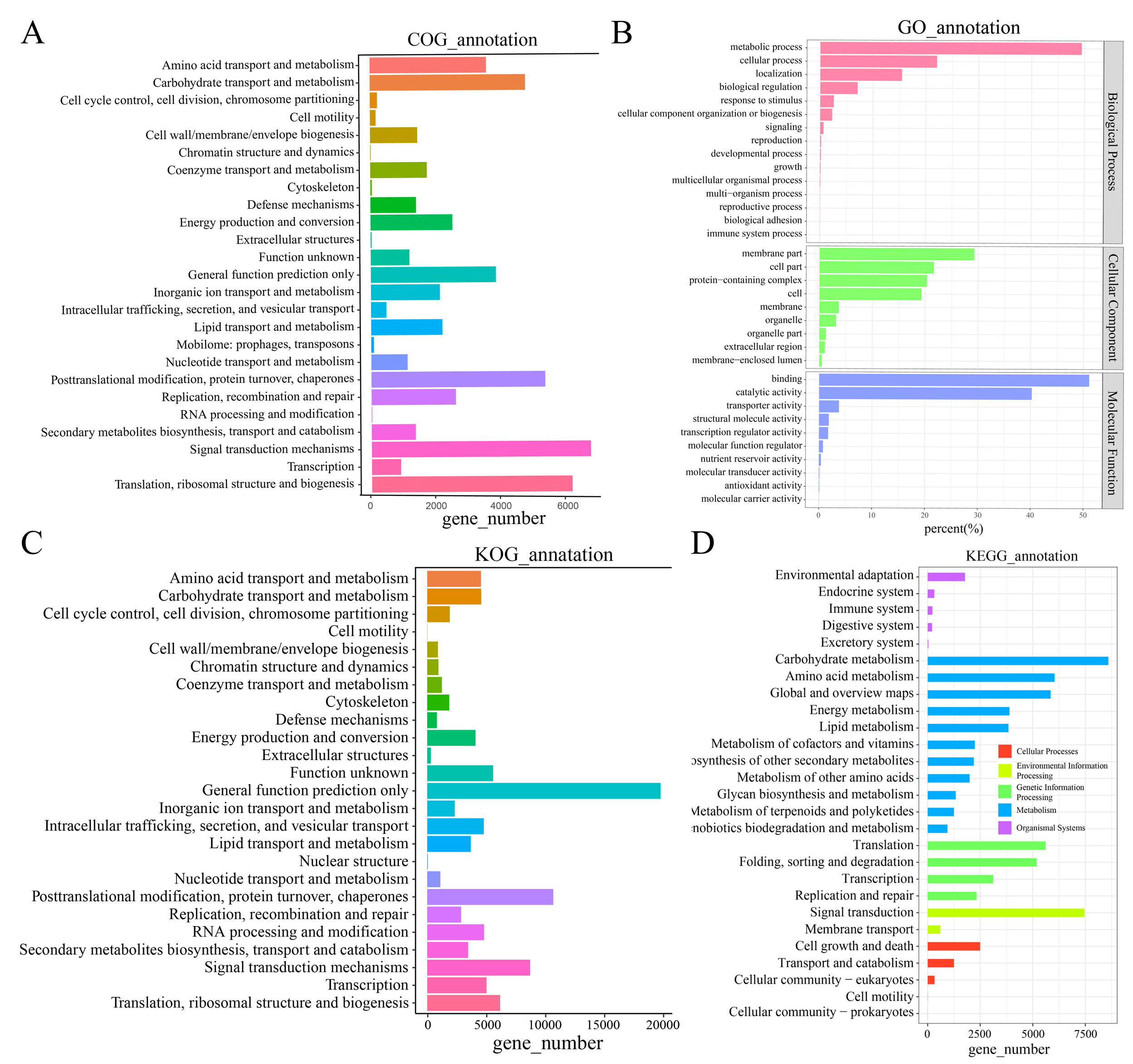


**Fig. S4 Protein functional annotation**

(A) Protein COG functional annotation; (B) Protein GO functional annotation; (C) Protein KEGG functional annotation; (D) Protein KOG functional annotation


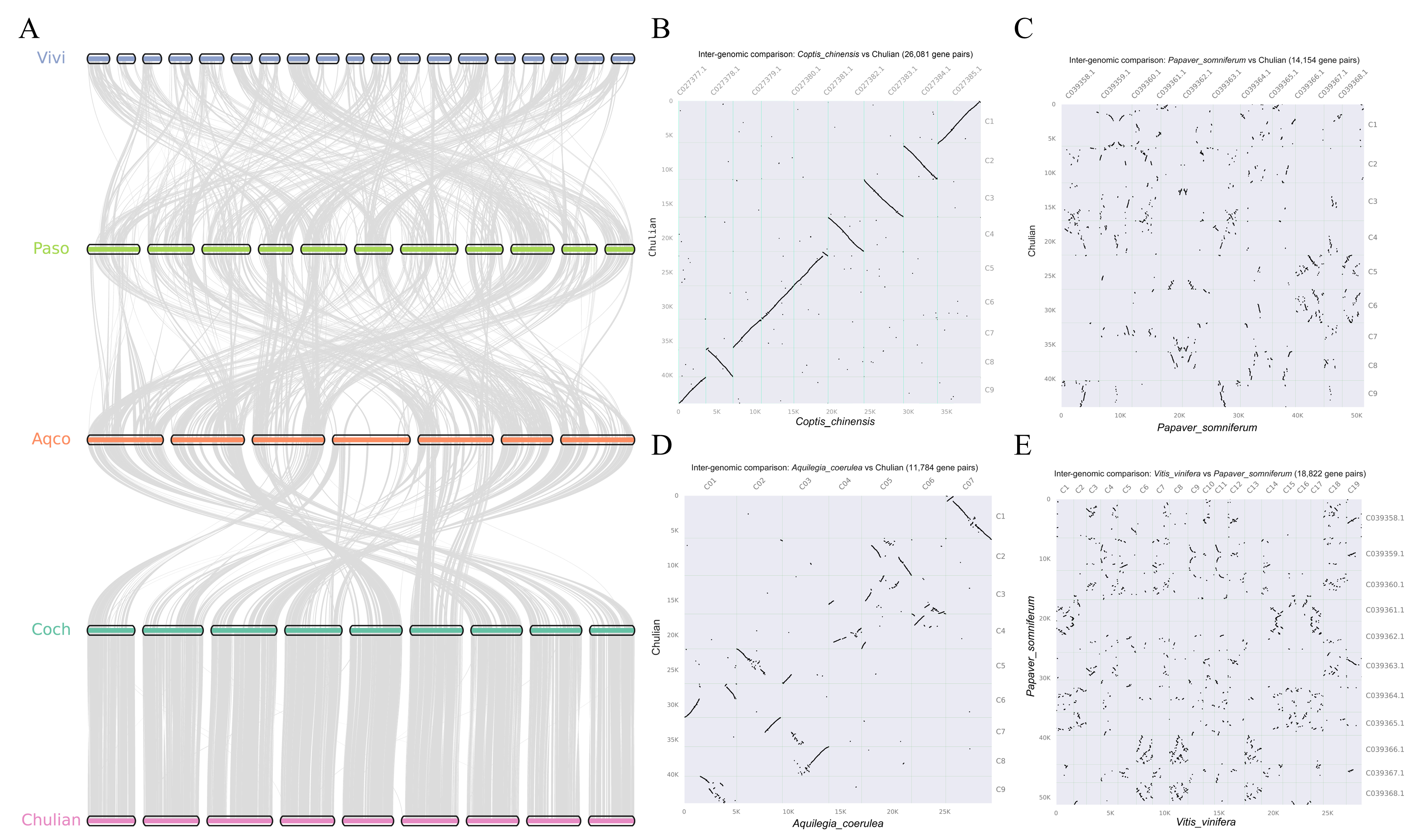


**Fig. S5** **The collinearity map of ‘Chulian No.1’ with other genomes**

(A) The genome of ‘Chulian No.1’ is collinear with *Coptis chinensis* (Liu et al,. 2021), *Aquilegia coerulea*, *Vitis vinifera* and *Papaver somniferum*; (B) Gene-based synteny analysis shown as a dot plot between ‘Chulian No.1’ and *Coptis chinensis* genomes; (C) Gene-based synteny analysis shown as a dot plot between ‘Chulian No.1’ and *Papaver somniferum* genomes; (D) Gene-based synteny analysis shown as a dot plot between ‘Chulian No.1’ and *Aquilegia coerulea* genomes; (E) Gene-based synteny analysis shown as a dot plot between *Vitis vinifera* and *Papaver somniferum* genomes


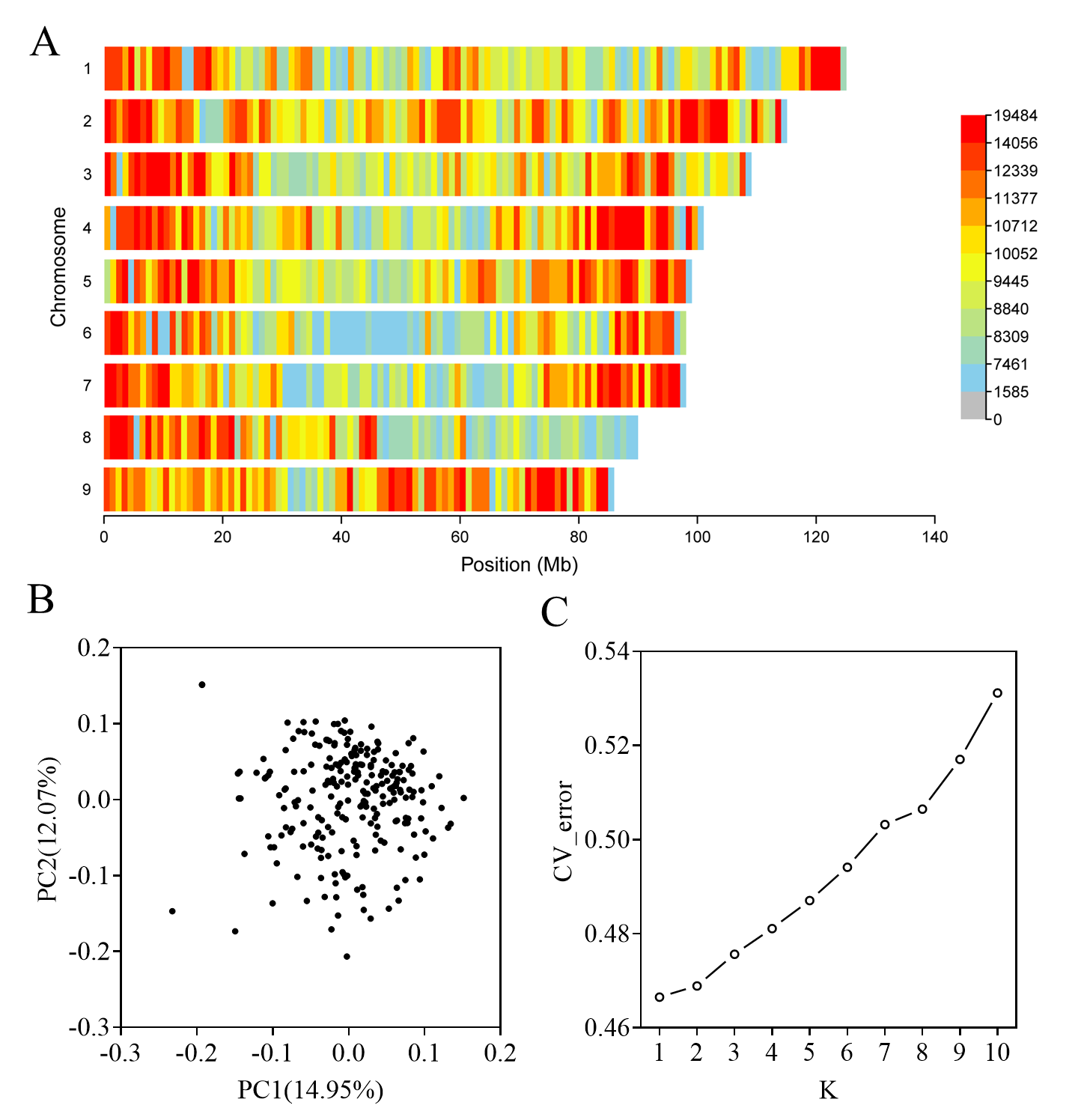


**Fig. S6 Population genetic analysis.** (A) Genome-wide SNP distribution across the *Coptis* population; legend: Number of SNPs per 1 MB on each chromosome. (B) PCA of genetic clustering patterns. (C) Cross-validation error curve for optimal subpopulation number determination.

**
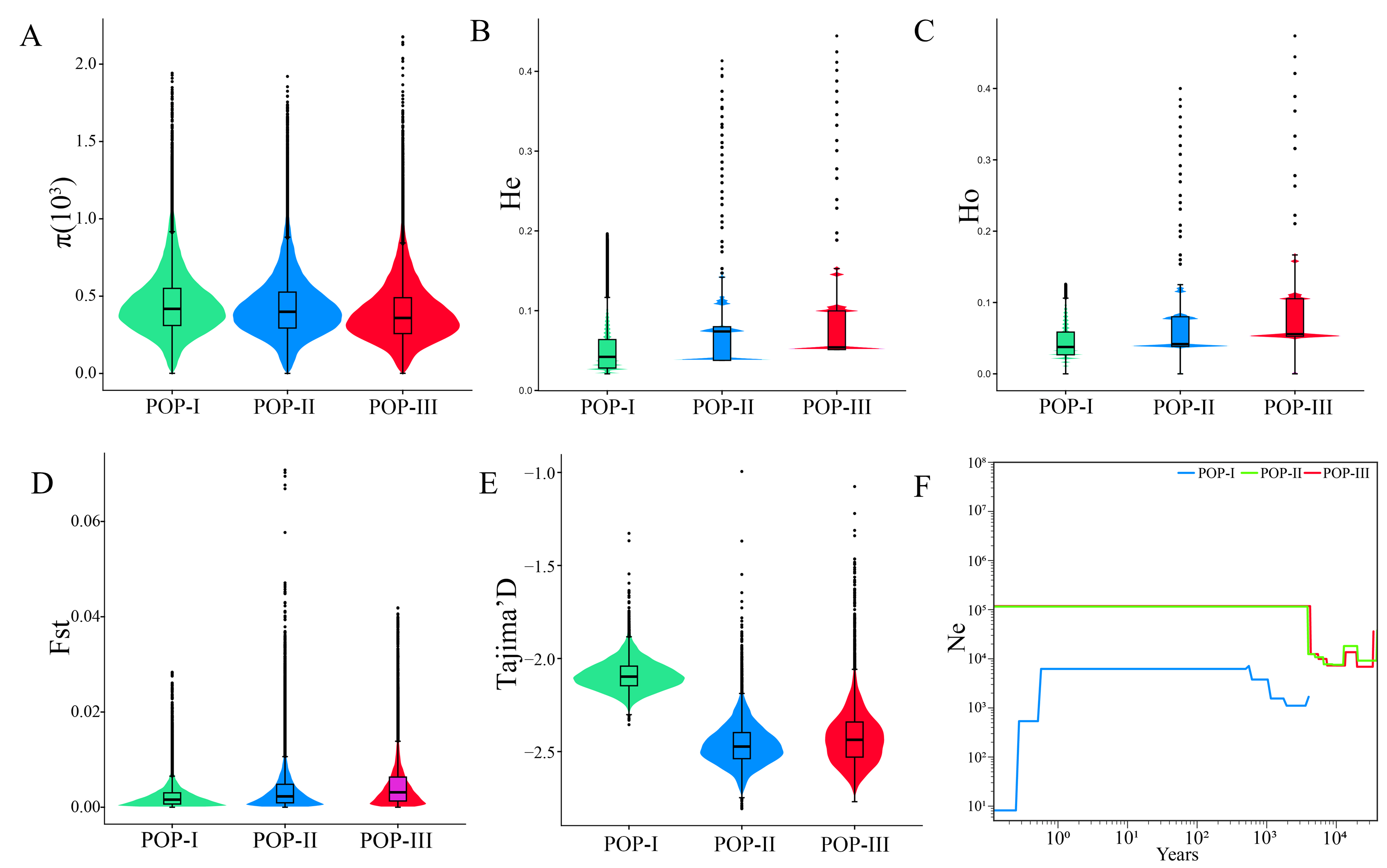
**

**Fig. S7 Integrated population genomic analyses reveal homogenized diversity under agroecological pressures.**

(A) Genome-wide nucleotide diversity (π ×10³, 100 kb sliding windows with 10 kb step size) across three geographically defined subpopulations: POP-I (red), POP-II (blue), POP-III (green). (B-C) Observed (Ho) versus expected heterozygosity (He). (D) Pairwise FST differentiation matrix (100 kb windows, 10 kb step). (E) Genome-wide Tajima’s D scan (100 kb windows). (F) Coalescent-based demographic reconstruction of effective population size (Ne) trajectories under mutation rate μ = 6.0 ×10⁻⁹ per site per generation.


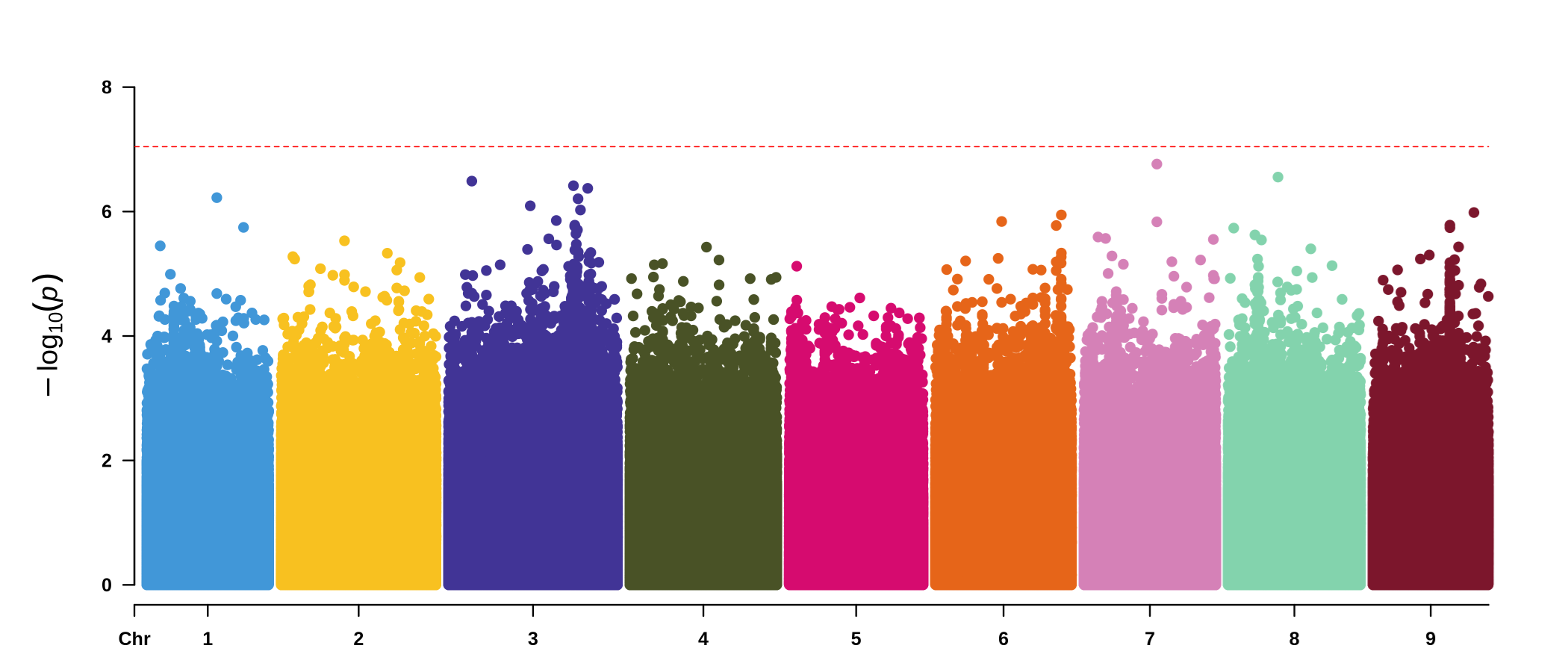


**Fig. S8 Genome-wide association study (GWAS) of leaf spot traits in *C. chinensis***

Manhattan plot displaying significant genetic associations across chromosomes (dots represent individual SNPs). The genome-wide significance threshold (red dashed line) corresponds to *P* = 1.29 × 10⁻⁸, calculated using (correction method, Bonferroni) for multiple testing. Peaks surpassing the threshold indicate loci strongly associated with leaf spot variation.


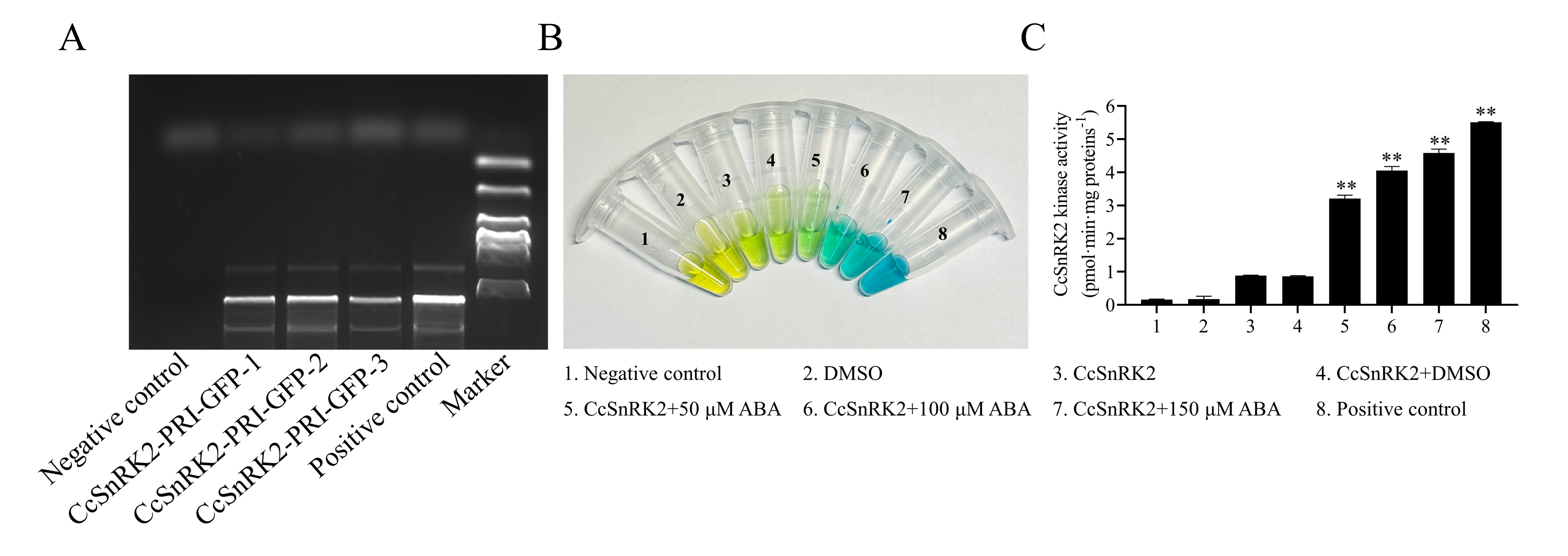


**Fig. S9 Transient expression and kinase activity assay of *CcSnRK2* in response to ABA.**

(A) Agarose gel electrophoresis confirming the presence of *CcSnRK2* gene in transiently transformed tobacco (*Nicotiana benthamiana*) leaves. (B) Visual comparison of kinase activity assays in *C. chinensis* leaves transiently expressing *CcSnRK2* under different ABA concentrations. (C) Quantification of *CcSnRK2* kinase activity under treatments 1-8. Bars represent mean ± SEM (n = 5 biological replicates). Asterisks indicate significant differences compared to *CcSnRK2* alone (**: p < 0.01, one-way ANOVA with Dunnett's post hoc test).
